# Differences in Biomechanical Determinants of ACL Injury Risk in Change of Direction Tasks Between Males and Females: A Systematic Review and Meta-Analysis

**DOI:** 10.1186/s40798-024-00701-z

**Published:** 2024-04-01

**Authors:** Thomas A. Donelon, Jamie Edwards, Mathew Brown, Paul A. Jones, Jamie O’Driscoll, Thomas Dos’Santos

**Affiliations:** 1 Section of Sport Section of Sport, Exercise and Rehabilitation Sciences, School of Human and Life Sciences, Canterbury Christ Church University, North Holmes Road, Canterbury, Kent, CT1 1Q UK; 2 School of Health Sciences, C702 Allerton Building, University of Salford, Salford, M6 6PU UK; 3 Department of Sport and Exercise Sciences | Manchester Metropolitan University, 2.01 Institute of Sport, 99 Oxford Road, Manchester, M1 7EL UK

**Keywords:** Cutting, Pivoting, Sex-comparison, Gender-comparison, Anterior-cruciate-ligament

## Abstract

**Background:**

Change of direction (COD) movements are associated with non-contact anterior cruciate ligament (ACL) injuries in multidirectional sports. Females appear at increased risk compared to males, which could be attributable to whole body kinematic strategies and greater multiplanar knee joint loads (KJLs) during COD which can increase ACL loading.

**Objective:**

The aim of this systematic review and meta-analysis was to examine and quantitatively synthesise the evidence for differences between males and females regarding KJLs and their biomechanical determinants (whole body kinematic strategies determining KJLs) during COD tasks.

**Methods:**

Databases including SPORTDiscus, Web of Science, and PubMed were systematically searched (July 2021–June 2023) for studies that compared differences in knee joint loads and biomechanical determinants of KJLs during COD between males and females. Inclusion criteria were: (1) females and males with no prior history of ACL injury (18–40 years); (2) examined biomechanical determinants of KJLs and/ or KJLs during COD tasks > 20°; (3) compared ≥ 1 outcome measure between males and females. Studies published between 2000 and 2023 examining a cutting task > 20° with a preceding approach run that compared KJLs or the whole body multiplanar kinematics associated with them, between sexes, using three-dimensional motion analysis.

**Results:**

This meta-analysis included 17 studies with a pooled sample size of 451 participants (227 males, 224 females). Meta-analysis revealed females displayed significantly less peak knee flexion during stance (SMD: 0.374, 95% CI 0.098–0.649, *p* = 0.008, I^2^: 0%); greater knee abduction at initial contact (IC) (SMD: 0.687, 95% CI 0.299–1.076, *p* = 0.001, I^2^: 55%); less hip internal rotation (SMD: 0.437, 95% CI 0.134–0.741, *p* = 0.005, I^2^: 34%) and hip abduction at IC (SMD: −0.454, 95% CI 0.151–0.758, *p* = 0.003, I^2^: 33%). No significant differences were observed between males and females for any internal or externally applied KJLs. All retrieved studies failed to control for strength, resistance training or skill history status.

**Conclusion:**

No differences were observed in KJLs between males and females despite females displaying greater knee abduction at IC and less peak knee flexion during the stance phase of CODs, which are visual characteristics of non-contact ACL injury. Further research is required to examine if this translates to a similar injury risk, considering morphological differences in strain characteristics of the ACL between males and females. This observation may in part explain the disproportionate ACL injury incidence in female multidirectional athletes. Further higher quality controlled research is required whereby participants are matched by skill training history, resistance training history and strength status to ensure an appropriate comparison between males and females.

## Key Points

- No differences between sexes were found for multiplanar KJLs despite previous research supporting this. Further research is required to identify if this is the case and if it affects injury risk between males and females.
- Females displayed greater knee abduction at initial contact, less peak knee flexion during stance, and less hip rotation and abduction at IC together with less peak hip flexion during stance during CODs.
- Previous research has failed to account for confounding factors such as skill training history, resistance training history and strength status together with gendered environmental factors that could contribute to the misinterpretation of COD differences between males and females.

## Background

Changes of direction (CODs) encompass an integral element of team sports and invasion games, which are associated with key decisive moments in sport, such as creating space, evading an opponent (i.e., tackle break success in rugby), and goal scoring [1]. Notational analysis has observed CODs to occur at frequencies of every four to six seconds across a number of sports such as netball [2], soccer [3] and hockey [4], with up to 700 CODs observed in soccer in a 90 min period [5–7]. Considering the rate and frequency of CODs in invasion games, it is of significant importance for coaching and sports medicine staff to condition athletes appropriately to meet these physical demands [8]. Although CODs are a key component of effective performance in multidirectional sports, COD actions are also a primary mechanism of non-contact ACL injury [9–11].

A substantial amount of anterior cruciate ligament (ACL) injuries are reported annually with an estimated global incidence of 68.6 per 100,000 person-years [12] alongside an observed increase in ACL reconstructions globally [12–15]. Despite recent advancements in sport technology, medicine and coaching practice, ACL injury rates are projected to rise [16]. Of clinical importance, 70% of ACL injuries are non-contact [17], with approximately 0.62 ACL injuries reported per 1000 player exposures [18]. It is of concern that a gynocentric pattern in the epidemiological literature is prevalent, with females being 1.7 times more likely to incur an ACL injury compared to male counterparts when matched for playing exposures [19], alongside a significantly greater risk of ACL re-injury [20]. ACL injuries are debilitating by nature, often requiring surgical intervention and a lengthy return to play time of 6–24 months [21, 22]. Substantial social and health related implications have also been identified following ACL reconstruction. These include a financial burden to public services (e.g., £63 million GBP spent on reconstructions annually in the United Kingdom [23]) associated with a decline in mental health [24]. An increased susceptibility to osteoarthritis has also been observed [25], coupled with 50% failing to return to sport within a year post-surgery [26]. The above necessitates a greater understanding of injury mitigation strategies in order to attenuate the financial, social and health related issues associated with ACL injury.

ACL ruptures occur when a catastrophic load is applied to the ACL, whereby the strain exceeds the ligament’s mechanical tolerance, resulting in tissue failure [27]. Previous cadaveric [28] and modelling [29–31] research have identified knee abduction, shear, and internal rotational loads (alternatively known as externally applied moments or torques) to substantially increase ACL strain independently, although observed strain is greatest when a combination of these loads is applied together [32]. Such have been termed multiplanar knee joint loads (KJLs) in the literature, and have been identified as a predictor of ACL injury [33] and also considered surrogate measures of non-contact ACL injury risk [34–36]. There is a propensity to generate large and potentially hazardous multiplanar KJLs during COD actions that are commonly performed in team sports, which are amplified with specific sub-optimal postures (e.g., knee abduction, extended knee posture, lateral trunk flexion), warranting their investigation in relation to non-contact ACL injury risk. A substantial amount of research has been undertaken in order to identify the biomechanical determinants of these KJLs in CODs to understand the kinetic, kinematics, and technical parameters associated with increased KJLs and potential non-contact ACL injury risk [33, 37–44]. This has provided practitioners with guidelines for optimal technique and how to mitigate KJLs in COD tasks [45], with researchers demonstrating a reduction in KJLs through targeted COD technique modification training interventions [37, 46, 47].

Females have been identified as exhibiting these sub-optimal body postures and greater relative KJLs during CODs more than their male counterparts [48–52]. This observation may provide some explanation of the disparity in reported injury rates. A previous systematic review and meta-analysis [53] identified females as displaying an increased knee abduction angle across a range of weight-bearing tasks, including running, landing and cutting. Knee abduction angle itself would only represent one component of the resultant knee abduction moment, and does not take into account the other segmental and kinetic contributions to multiplanar KJLs [36]. To date, two systematic reviews have examined differences between males and females in biomechanical surrogates of non-contact ACL injuries in landing [54] and cutting [55]. Benjaminse et al. [55] identified greater knee abduction angles in two of their retrieved studies (one with a large effect size (ES) of 0.99), and knee abduction moments, whilst Beaulieu et al. [54] identified females exhibited greater knee abduction angles during landing. A limitation of both these systematic studies is that they did not meta-analyse the data to examine and establish the magnitude of differences between males and females. Meta-analysis would previously not have been possible in the aforementioned review [55], due to insufficient literature available (seven retrieved studies from the years 1947–2008). In recent years there has been a substantial research effort to further understand biomechanical differences between males and females during cutting [50, 51, 56–64], allowing for meta-analysis to be undertaken.

Therefore, the aim of this systematic review and meta-analysis is threefold: firstly, to synthesise the evidence regarding differences between males and females regarding KJLs and their biomechanical determinants during CODs measured through 3-dimensional (3D) motion (and ground reaction force [GRF]) analysis; secondly, to identify relevant effect modifiers in uninjured athletes and thirdly, to provide recommendations and directions for future research examining sex differences in COD tasks. It was hypothesised that females would exhibit less knee flexion and greater knee abduction angles, and greater knee abduction moments during COD tasks. The findings of this meta-analysis may assist in ACL injury mitigation strategies, injury screening protocols, and physical preparation and management of female and male athletes.

## Methods

A systematic review and meta-analysis were performed in accordance with the recently updated PRISMA (Preferred Reporting Items for Systematic Reviews and Meta-Analyses) guidelines [65]. The study was also registered with PROSPERO on 17th November 2021 (CRD42021266215) and adhered to the ethical recommendations for the publishing of systematic reviews in accordance with Wager and Wiffen [66]. Some minor amendments were made to the review completion date and inclusion criteria; namely, the population element of the Population, Intervention, Comparator, Outcomes and Study design (PICOS) framework was amended to include healthy and recreationally active participants and removed the performance level stipulation of playing twice a week in given sport. The rationale for amendment was due to the majority of retrieved papers failing to specify competition playing frequency, and a paucity of literature examining elite athletes [67]. The registration document was amended to reflect these changes appropriately (4th October 2022).

### Study Inclusion and Exclusion Criteria

A PICOS framework was constructed to define the inclusion and exclusion criteria for this study and is presented in Table 1. This article is part of a wider project and search strategy identifying a number of neuromuscular and biomechanical differences between sexes in COD tasks. It was decided to solely focus on biomechanical injury risk surrogates obtained from 3D motion analysis, with the neuromuscular surrogates and data obtained from modelling and simulation to be disseminated in another project. The aim of this paper is to analyse the sex differences in biomechanical surrogates of non-contact ACL injury risk (biomechanical determinants of KJLs and KJLs themselves) obtained from 3D motion and GRF analysis.

**Table 1:** PICOS framework used to define inclusion and exclusion criteria of studies

Population	For the purpose of this review, male, female and sex refers to individuals assigned male or female at birth based on biological characteristics Healthy women – No restrictions were placed regarding reproductive status and hormonal contraception (HC) usage Healthy males (as a control for sex comparison of biomechanics) Age 18–40 years (children and pre/adolescent populations were excluded to control for the effect of puberty or changes during adolescence) Competing in or familiar with jump landing / cutting dominant, field / court based invasion games / sports Performance level: Tier 1 minimum – Tier 5 [67]—Healthy, recreationally active and elite athletes No history of ACL injury
Intervention/method	A specific intervention was not investigated but participants were required to meet the population criteria above Studies must have examined biomechanical surrogates of non-contact ACL injury risk injury during pre-planned or unplanned change of direction tasks, with 3D motion and/or GRF analysis (inclusive of pre and/or unplanned tasks) Studies which adhered to the following change of direction task criteria were included: A preceding approach run of a minimum of 3 steps containing a subsequent change in direction > 20° The decision was made to omit tasks that included a split/ false step or hop or that omitted an approach run in line with previous COD definitions [68, 69] Omission of an approach run would not truly replicate the loading parameters of non-contact ACL injury situations due to the absence of a deceleration (as deceleration has been identified as the component where most noncontact ACL ruptures occur) (Donelon et al., 2020 [45])
Comparator	To determine the effect of sex: A direct between group comparison of a biomechanical surrogate of non-contact ACL injury risk between females and males (acting as a control)
Outcome	Precise mean and SD provided for injury risk factors between males and females Biomechanics: Knee abduction, rotation, flexion moments / impulse (knee joint loads) [45, 70–74] Proximal anterior tibial shear [75] Technical, kinetic, or kinematic determinants of surrogates of injury risk (knee joint loads) [36, 70, 76] at initial contact (IC) (first instance of ground contact in COD) and peak value obtained during stance (across the full cutting cycle 0–100%) related to quadriceps, ligament, trunk, and leg dominance, such as: Vertical/posterior GRF/ impulse *Initial or peak* Lateral trunk flexion/rotation angle Hip internal rotation angle Knee valgus / internal rotation angle Knee flexion / hip flexion Foot progression angles Rearfoot/ forefoot strike Coordination changes Asymmetries
Study design	Peer review full article in English, examining humans from the year 2000 onwards due to a lack of 3D motion analysis research before this date Direct assessment of change of direction biomechanics (with surrogates of injury) between healthy males and females
Other data extraction	The following data were extracted and recorded in a spreadsheet: (1) Author names, publication year and country of origin (2) Sample size and participant characteristics including sport(s), playing level/status, training history, strength history/status/profile, reproductive status(females), hormonal contraception usage (3) Angle of COD task (4) Anticipatory nature of COD task (planned/unplanned) (5) If unanticipated, method and timing of stimulus presentation (6) Approach velocity prior to COD (7) How approach velocity was controlled for / calculated (8) Rest period between trials (if stated) (9) How risk factor (ACL surrogate) was assessed (methods) (10) Reliability and familiarisation stated for outcome measures / tasks (11) Outcome measures (mean, SD,) (12) Any other empirical data available for a variable that could mitigate any sex differences in surrogate injury risk identified (e.g. strength or experience / playing time) (13) For female populations, information relating to: a. Reproductive status b. Menstrual cycle phase c. Hormonal contraception use

ACL—Anterior Cruciate Ligament, COD—Change of Direction, GRF—Ground Reaction Force, HC—Hormonal Contraception, MSK—Musculoskeletal, SD—Standard Deviation

In randomised controlled trials or studies examining the effect of an intervention such as bracing [59] or fatigue [58, 77], only baseline data from the control group were extracted for subsequent analysis. Exclusion criteria consisted of studies examining injured or ACL reconstructed populations, case studies, and poster presentations/ conference proceedings. Studies that did not meet the PICOS criteria were excluded from the review.

### Search Strategy

A literature search was performed using PubMed, Web of Science, and SPORTDiscus databases from July 2021 to June 2023 with the final search date of 1st June 2023. A schematic of search methodology in accordance with established PRISMA guidelines [44] is presented in the results below. Search terms that were used are presented in Table 2:

**Table 2:** Search strategy used for literature searching

Search strategy	
“Anterior cruciate ligament” NOT reconstruction OR “ACL” NOT reconstruction	AND
“Sex-differences”, OR “gender-differences” OR “Sex-comparison” OR “gender-comparison” OR “sex” OR “gender”	AND
“biomechanics” OR “biomechanical-determinants” OR “technical-determinants” OR “kinetics” OR “kinematics” OR “Neuromuscular” OR “electromyography” OR “muscle activation”	AND
“Change of direction”, or “Cutting manoeuvre”, or “Run and cut”, or “Run-and-cut”, or “Sidestepping”, or “Side-stepping” or “Shuttle-run”	AND

Articles retrieved by this search were then title, abstract and then full-text screened against the PICOS framework to examine their suitability for inclusion by the lead researcher (TD) and another researcher (TDS). Should disagreement have arisen surrounding the inclusion of an article, a third researcher (PJ) was consulted and their decision deemed final. Bibliographies of prospectively eligible (full texts reviewed) were then hand searched in order to identify further eligible studies.

### Methodological Quality and Publication Bias

An assessment of methodological quality was independently undertaken by two of the researchers (TD and TDS) as per previously established methods [70, 78, 79] using a COD specific scale constructed by Brown et al. [80]. This is deemed to be more suitable for assessing the methodological quality of COD studies due to the omission of criteria such as random allocation, assessor blinding and subject blinding that are present in more commonly used scales such as the Cochrane or Delphi, Physiotherapy, Evidence Database scales [79, 81]. Change of direction specific protocols were rigorously assessed by the tool due to specific criteria present, such as the allowance of practice trials, duration of rest between trials and velocity of COD tasks. Each component was individually scored from 0 to 2 (where 0 = clearly no, 1 = maybe or insufficient information; and 2 clearly yes). Any disagreement was resolved through consensus and discussion involving a third researcher (PJ). The methodological assessment tool is presented in Table 3.

**Table 3:** Brown assessment of methodological quality

Question	Criteria	Beaulieu et al. [56]	Condello et al. [57]	Iguchi et al. [58]	Ihmels et al. [59]	Khalid et al. [60]	McLean et al. [49]	McLean et al. [48]	McLean et al. [88]	Nagano et al. [61]	O’Connor et al. [62]	Pollard et al. [89]	Pollard et al. [86]	Schreurs et al. [63]	Sigward and Powers 2006 [52]	Sigward et al. [51]	Sigward et al. [50]	Tanikawa et. [64]
1	Power analysis was performed and justification of study sample size	0	0	1	2	0	2	2	2	2	2	2	2	2	0	2	0	2
2	Athlete demographics were clearly defined: gender, age, body height, and body mass at time of test	2	2	2	2	2	2	2	2	2	2	2	2	2	2	2	2	1
3	Athlete characteristics were clearly defined: sport, experience or activity level and level of play	2	2	1	1	2	0	2	2	1	2	2	2	1	2	2	2	1
4	Inclusion and exclusion criteria were clearly stated for athletes	0	0	0	2	0	2	2	2	2	2	2	2	2	2	2	2	2
5	Proper training and practice trials of the test were given to the athletes allowing for adequate familiarisation	2	2	2	0	2	0	0	0	2	2	2	2	2	2	0	0	1
6	Methods were described in great detail to allow replication of the test. Testing devices, n of trials, n and duration of rest, speed, angle of COD	2	2	1	1	2	1	1	1	1	1	2	1	2	1	1	1	1
7	Test–retest reliability of measurement device reported	0	1	0	0	0	0	0	0	1	0	0	0	0	2	0	0	0
8	Outcome variables clearly defined	2	2	2	2	2	2	2	2	2	2	2	2	2	2	2	2	1
9	Statistical analyses were appropriate	2	2	1	1	1	2	2	2	2	1	2	2	1	2	1	1	1
	Total score (maximum 18)	12	13	10	11	12	11	12	13	15	15	16	15	14	15	11	10	10
	Score as %	67%	72%	56%	61%	67%	61%	67%	72%	83%	83%	89%	83%	78%	83%	61%	56%	56%

COD—Change of Direction, n—number

### Data Extraction

The following data were extracted by the lead author in Table 4 (TD): quantitative data pertaining to study methodology, participant characteristics (age, height, mass) and verification, biomechanical ACL injury risk surrogates during initial contact (IC) (defined as the first instance of foot-contact during the cut), range of motion (ROM; defined as point of IC to maximum knee flexion) and peak stance (defined as the peak value obtained across 100% of the cutting cycle), reliability measures and measured outcomes and results (means and SDs of both male and female conditions). Once extracted, data were pooled together for COD angle and anticipation status of the COD task due to insufficient data for separate angle-dependent and anticipatory analysis. The authors acknowledge that anticipated and unanticipated COD conditions have been identified as producing significantly different outcome metrics, although sex differences in kinematics and KJLS have been observed under both anticipated [48, 52] and unanticipated [50, 56] conditions. This is attributable to a time constraint to orientate the body in preparation for the COD and therefore the biomechanical demands to complete the task remain the same [82]. This decision was made based on the above and considering the aim of this meta-analysis is to identify differences between males and females in COD tasks. Data were then systematically separated by variable timing (IC, ROM or during peak stance), moment convention (internal / external) and reference frame prior to analysis.

**Table 4:** Variables extracted from retrieved studies

Outcome measures	Precise mean and SD provided for injury risk factors between males and females Biomechanics: Knee abduction, rotation, flexion moments / impulse (knee joint loads) [70–74] Proximal anterior tibial shear [75] Technical, kinetic, or kinematic determinants of surrogates of injury risk (knee joint loads) [36, 70, 76] at initial contact (IC) (first instance of ground contact in COD),range of motion (ROM; defined as point of IC to maximum knee flexion) and peak stance (peak value obtained during the full cutting cycle 0–100%) related to quadriceps, ligament, trunk, and leg dominance, such as: Vertical / posterior GRF/ impulse *Initial or peak* Lateral trunk flexion/rotation angle Hip internal rotation angle Knee valgus / internal rotation angle Knee flexion / hip flexion Foot progression angles Rearfoot/ forefoot strike Coordination changes Asymmetries
Other data extraction	The following data were extracted and recorded in a spreadsheet: (1) Author names, publication year and country of origin (2) Sample size and participant characteristics including sport(s), playing level/status, training history, strength history/status/profile, reproductive status(females), hormonal contraception usage (3) Angle of COD task (4) Anticipatory nature of COD task (planned/unplanned) (5) If unanticipated, method and timing of stimulus presentation (6) Approach velocity prior to COD (7) How approach velocity was controlled for / calculated (8) Rest period between trials (if stated) (9) How risk factor (ACL surrogate) was assessed (methods) (10) Reliability and familiarisation stated for outcome measures / tasks (11) Outcome measures (mean, SD,) (12) Any other empirical data available for a variable that could mitigate for any sex differences in surrogate injury risk identified (e.g. strength or experience / playing time?) (13) For female populations, information relating to: a. Reproductive status b. Menstrual cycle phase c. Hormonal Contraception use

ACL—Anterior Cruciate Ligament, COD—Change of Direction, GRF—Ground Reaction Force, HC—Hormonal Contraception, MSK—Musculoskeletal, SD—Standard Deviation

All variables that were extracted are present in Table 4.

### Statistics

All statistical analysis was performed using Comprehensive Meta-Analysis (Comprehensive Meta-Analysis Version 3, Biostat, Englewood, NJ, USA). Separate pooled analyses for initial contact and peak variables obtained during stance were performed for each independent parameter to establish the standardised mean difference (SMD) between male and female groups. SMD was selected as the appropriate outcome measure due to inter-study variance in data reporting approaches. SMD effect thresholds were as follows: 0.2–0.5 small effect, 0.5–0.8 medium effect and above 0.8 as a large effect [83]. Where sufficient data were available, subgroup analyses were also performed on COD angle, anticipation, and sport. Further meta-regression moderator analyses were performed on methodological quality, age, mass, achieved velocity and playing experience. Statistical heterogeneity was assessed alongside the pooled analysis and reported as the I^2^ statistic. The application of fixed or random effects analysis was determined by the absence or presence of significant statistical heterogeneity. The threshold for heterogeneity significance was set at an I^2^ statistic of > 40% in which an Egger's regression test was systematically planned to assess the presence of funnel plot asymmetry to account for potential publication bias [84]. Pooled analysis results were considered significant if *p* < 0.05 and the Z-value > 2.

## Results

### Search Results

**Fig. 1:**
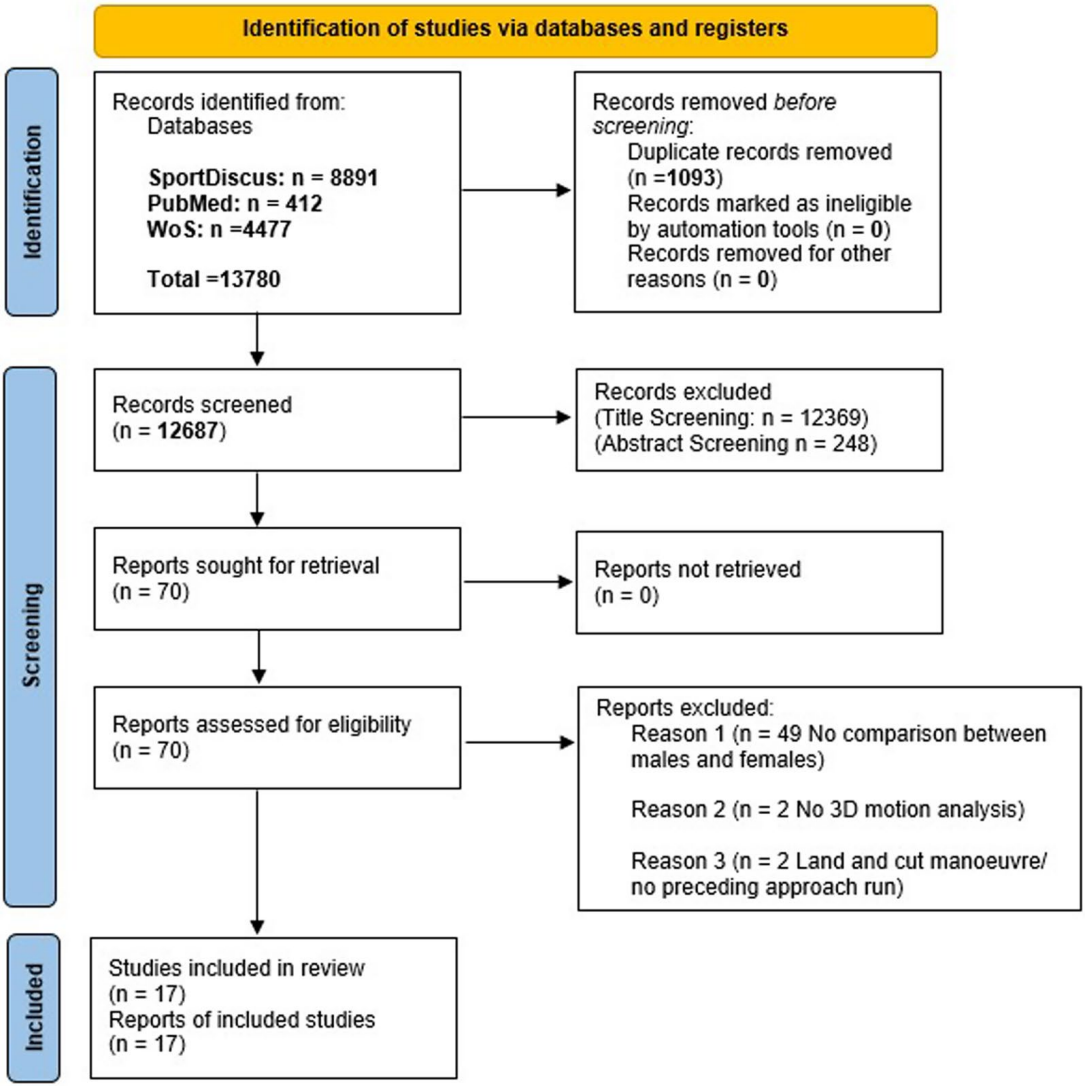
Flow chart illustrating the different phases of the search strategy and study selection process. Key: 3D—Three dimensional, WoS—Web of Science

 Figure 1 presents a flow chart summarising the results of the systematic search process. Following duplicate removal and application of eligibility criteria, 14 studies [48–51, 57–62, 64, 85–88] were initially included for meta-analysis. Following reference list screening of eligible studies, a further three studies were deemed eligible for inclusion [52, 63, 89], resulting in 17 studies overall used for quantitative analysis. The authors had access to the full text for all included studies.

### Characteristics of the Studies

The 17 included studies (Table 5) evaluated sex differences in surrogates of non-contact ACL injury risk. A total of 451 participants were included in the analysis (227 males, 224 females). The mean (SD) age, height and mass of males and females were 21.10 (1.74) years, 1.79 (0.05) m, 74.5 (6.84) kg and 20.82 (1.70) years, 1.67 (0.05) m and 62.83 (6.55) kg, respectively. In total, 254 participants played soccer (132 males and 122 females) and 109 were defined as physically active or recreationally active (54 males, 55 females). Forty participants played basketball (20 males 20 females) whilst 29 (13 males, 16 females) were of a non-defined team sporting background [63]. Finally, 16 participants were termed “proficient” [49] in cutting manoeuvres in addition to 3 female lacrosse players used in a study [61]. Mean sporting experience in years was stated in 11 studies [48, 51, 52, 56, 57, 60, 62, 86–89] and was 12.18 years (2.58) for males and 11.53 (2.91) for females. Only one study [56] controlled for menstrual cycle phase in female participants. No study reported resistance training history, hormonal contraceptive use or reproductive status. Thirteen studies [50–52, 56–58, 60–64, 86, 89] reported familiarisation prior to data collection consisting of numerous practice trials [50–52, 57, 58, 61, 63, 64, 86] or a specific session delivered separately [56, 62, 77, 89]. Only two studies [52, 57] reported reliability statistics for their outcome measures, namely intra-class correlation coefficients (ICCs) [57], and coefficients of multiple correlations (CMCs) [52].

**Table 5:** Summary of study characteristics

Study	Participants and classification [67]	COD task	Familiarisation/reliability	Velocity (m.s)	Outcomes	Comments
Beaulieu et al. [85]	15 Female: (21.1 ± 3.6 years, 168.3 ± 5.3 cm, 62.4 ± 4.9 kg) 15 Male: (22.9 ± 3.7 years, 178.2 ± 8.0 cm, 75.1 ± 6.7 kg) Elite soccer players (Tier 4/3)	45° UA cut	Separate practice/familiarisation session	4.0–5.0	3D Kinematics (Sagittal, frontal, transverse) 3D kinetics (Sagittal, frontal, transverse) Peak values and at IC	Only study to control for MC phase: Tested all females in follicular phase of MC
Condello et al. [57]	12 Females: (21.0 ± 2.7 years, 166.9 ± 4.1 cm, 61.9 ± 6.2 kg) 14 Males: (19.6 ± 1.5 years, 178.9 ± 4.7 cm, 73.1 ± 7.2 kg) NCAA Div III soccer players (Tier 3)	60° PP cut (each limb)	Submaximal trials ICCs of outcome variables	Not stated	vGRF, mlGRF, contact time, Performance cutting angle, minimum horizontal distance (foot plant width)	Only kinematics and forces measured
Iguchi et al. [58]	12 Females: (21.9 ± 1.2 years, 160.6 ± 5.0 cm, 55.0 ± 5.2 kg) 11 Males (22.9 ± 1.0 years, 170.9 ± 4.5 cm, 64.2 ± 7.6 kg) “Recreationally active” (Tier 1)	60° UA cut	5 PP familiarisation trials	3.0	3D hip and knee kinematics (Sagittal) GRF impulse (50 ms, 100 ms)	CMJ fatigue eliciting protocol Pooled intervention conditions when performing sex comparisons
Ihmels et al. [59]	17 Females: (21.6 ± 2.8 years, 1.7 m ± 0.1, 64.6 ± 7.4 kg) 17 Males: (22.3 ± 3.3 years, 1.8 ± 0.1 m, 78.8 ± 10 kg) “Recreationally active” (Tier 1)	45° PP cut	None	4.0	Peak 3D knee kinematics and kinetics (Sagittal, Frontal and Transverse) Peak 3D ankle kinetics and ROM (Sagittal and Frontal)	Sex differences only observed in women wearing prophylactic braces and not control All conditions were pooled for subsequent sex analysis
Khalid et al. [60]	6 Females (19.33 ± 1.97 years, 1.61 ± 0.04 m, 55.4 ± 9.56 kg) 6 Males (20.17 ± 1.83 years, 1.74 ± 0.02 m, 66.58 ± 6.00 kg) Collegiate soccer players (Tier 2)	45° PP and UA cuts	1 h familiarisation session delivered within 3 days of testing	4.5–5.5	3D kinetics (Sagittal, frontal, transverse) Peak values	Small sample size of 6 Males and 6 Females Fatigue data pooled when performing sex comparisons
McLean et al. [49]	8 Females: (23.2 ± 3.8 years, 167.3 ± 6.5 cm, 64.1 ± 5.0 kg) 8 Males: (23.2 ± 3.8 years, 177.5 ± 8.3 cm, 73.1 ± 3.8 kg) “Proficient in sidestep manoeuvres”	PP 30–40° cut	Not reported	4.5–5.5	Peak 3D Hip and knee kinematics (Sagittal, frontal and transverse) Rearfoot pronation mGRF	No joint moments examined
McLean et al. [48]	10 Females: 21.1 ± 3.0 years, 176.0 ± 11.1 cm, 76.1 ± 12.4 kg 10 Males: 20.2 ± 1.9 years, 184.7 ± 8.0 cm, 81.9 ± 9.8 kg NCAA DIV I Basketball players (Tier 4)	35–55° path demarcated intended for PP 45° cut	Not reported	4.5–5.5	Peak 3D Hip and Knee Kinematics (Sagittal, frontal, transverse); 3D ankle Kinematics (sagittal and frontal) Peak 3D knee kinetics (Frontal)	Only frontal plane kinetics examined
McLean et al. [88]	10 Females: 21.1 ± 3.0 years, 176.0 ± 11.1 cm, 76.1 ± 12.4 kg 10 Males: 20.2 ± 1.9 years, 184.7 ± 8.0 cm, 81.9 ± 9.8 kg NCAA DIV I Basketball players (Tier 4)	35–55° path demarcated intended for PP 45°cut and PP 180°	Not reported	4.5–5.5	Peak and IC: 3D Hip and Knee Kinematics (Sagittal, frontal, transverse) 3D ankle Kinematics (sagittal and frontal)	No measurement of kinetics
Nagano et al. 2011 [61]	10 Female (7 Soccer 3 Lacrosse) 20.1 ± 1.4 years, 1.61 ± 0.06 m, 56.8 ± 7.4 kg 10 Male (Soccer)20.7 ± 1.3 years, 1.75 ± 0.05 m, 66.9 ± 6.2 kg, “Cutting sports”	PP 180° turn	“Several preparation trials”	“Maximal”	3D knee kinematics (sagittal, frontal, transverse) at IC and 75 ms and 150 ms 3D trunk kinematics: (sagittal and frontal) at IC and 75 ms and 150 ms	No knee moments calculated
O’Connor et al. [62]	17 Females: 20.9 ± 1.5 years, 1.68 ± 0.06 m, 62.9 ± 5.9 kg 16 Males: 22.7 ± 2.7 years, 1.81 ± 0.08 m, 86.1 ± 13.5 kg “Recreationally active” (Tier 1)	5 trials of UA 45° cut of dominant leg	Separate familiarisation session delivered one week prior to main testing	4.5–5.0	3D knee kinematics at IC and ROM (sagittal, frontal and transverse planes) 3D kinetics (internal joint moments; sagittal, frontal and transverse)	Multiple dynamic conditions pooled together prior to performing sex comparison
Pollard et al. [89]	12 Females: 19.3 ± 1.1 years, 1.66 m ± 0.05 m, 62.5 ± 6.9 kg 12 Males 19.7 ± 1.5 years 1.80 ± 0.07 m, 76.1 ± 5.9 kg NCAA Collegiate soccer players (Tier 3)	7–8 trials of UA 45° right legged cut	Practice session including several Preplanned and unanticipated trials	5.5–6.5	Peak 3D hip and knee Kinematics (Frontal and Transverse) Peak 3D hip and knee kinetics (Frontal and Transverse)	No sagittal angles and kinetics calculated despite their association with KJLs
Pollard et al. [86]	15 Females: 19.4 ± 1.5 years, 167.4 ± 8.0 cm, 65.9 ± 7.0 kg 15 Males: 19.6 ± 1.9 years, 179.1 ± 6.0 cm, 74.2 ± 7.0 kg NCAA Collegiate Soccer Players (Tier 3)	4 trials of 5 m right legged PP 45° cut	3–5 practice trials	5.5–7.0	Average 3D hip kinematics (Sagittal Frontal and transverse) Peak hip kinetics (Sagittal Frontal and transverse)	Only examined hip kinematics and kinetics
Schreurs et al. [63]	16 Females: 21.9 ± 2.5 years, 173.6 ± 6.9 cm and 67.1 ± 7.1 kg 13 Males: 22.5 ± 2.3 years, 190.2 ± 7.7 cm and 80.1 ± 10.3 kg “Team sport athletes”	10 trials of PP maximal 45°, 90°, 135°, and 180° CODs	None reported	“Maximal”	3D knee kinematics and ROM (Sagittal) 3D knee kinetics (Sagittal and Frontal)	Pooled COD conditions to perform sex analysis Only knee examined Measures reported at instance of peak valgus
Sigward and Powers 2006 [52]	15 Females: 19.4 ± 1.5 years, 167.4 ± 8.0 cm, 65.9 ± 7.0 kg 15 Males: 19.6 ± 1.9 years, 179.1 ± 6.0 cm, 74.2 ± 7.0 kg NCAA div I or II Soccer Players (Tier 3 and 4)	4 trials of 5 m right legged PP 45° cut	“Practice trials” Test–retest reliability reported	5.5–7.0	3D knee kinematics (Sagittal, frontal and transverse) 3D knee kinetics (Moment and moment impulse) (Sagittal frontal and transverse)	Only study to report test–retest reliability
Sigward et al. [51]	20 Females: 19.7 ± 0.2 years, 167.0 ± 1.6 cm, 51.8 ± 1.7 kg 20 Males: 19.7 ± 0.2 years, 181.1 ± 1.6 cm, 78.6 ± 1.7 kg NCAA div I or II Soccer Players (Tier 3 and 4)	4 trials of UA 45° cut with 7 m approach	“Practice trials”	4.5–5.5	Peak 3D Kinematics (frontal) Peak 3D Kinetics (Frontal internal) vGRF, pGRF, lGRF	Pooled maturation data to conduct sex comparisons
Sigward et al. [50]	20 Females: 18.25 ± 2.15 years, 1.63 ± 0.07 m, 59.55 ± 7.38 kg 25 Males: 18.76 ± 2.09 years, 1.80 ± 0.07 m, 75.58 ± 8.02 kg NCAA div I or II Soccer Players (Tier 3 and 4)	4 trials of UA 45° and 110° cuts with 7 m approach	“Practice trials”	4.5–5.5	3D Hip kinematics (Frontal and Transverse) 3D kinetics (Frontal) vGRF, pGRF, lGRF	Pooled data for angles to conduct sex comparisons
Tanikawa et al. [64]	Pooled male and female: 25.4 ± 3.5 years, 1.67 ± 0.1 m, 60.2 ± 10.1 kg	2 trials of 90° cut at self-selected speed	1 practice trial	Self-selected	3D knee kinematics and kinetics (Sagittal, frontal and transverse)	Trials performed barefoot and exceptionally low approach velocity (2.10 m.s)

°–degrees, 3D—Three-dimensional, cm—centimetre, CMJ—countermovement jump, COD—change of direction, GRF—ground reaction force, IC—initial contact, ICC—intra-class correlation coefficient, kg—kilogram, KJL—knee joint loads, lGRF—lateral ground reaction force, MC—menstrual cycle, mGRF— medial ground reaction force, ms—milliseconds, pGRF—posterior ground reaction force, PP—Preplanned, ROM—range of motion, UA—unanticipated, vGRF—vertical ground reaction force

Nine studies utilised a 45° COD task [48, 51, 52, 56, 59, 62, 77, 86, 89], with two adopting a 60° sidestep [57, 58]. Two studies adopted multiple COD angles including 45° and 110° [50], 45° and 180° [88], and 45–180° in four 45° increments [63]. One study each used isolated 90° [64] and 180° [61] CODs. Ten of the included studies included an anticipated COD task [48, 49, 52, 57, 59, 61, 63, 64, 86, 88]; with one study examining both unanticipated and anticipated CODs [77]. Of the six studies utilising unanticipated conditions [50, 51, 58, 62, 85, 89], a task choice approach was utilised requiring a decision between a 45°, 110° or straight run [50, 51]; a 30° crossover cut and 60° sidestep [58]; a straight-line run, “hard stop” [62, 85] or stop jump [89].

### Assessment of Methodological Quality

Assessment of methodological quality is presented in Table 3. The mean score for the Brown Methodological Quality of Assessment for the 17 included studies was 12.65 (70.26%) ± 1.97 (10.94%). Scores ranged from 10 (55.5%) [50, 58, 64] to 16 (88.8%) [89]. Nine studies were below this mean score [48–51, 56, 58, 59, 64, 77] with eight studies presenting greater methodological quality greater than the mean [52, 57, 61–63, 86, 88, 89]. Between day reliability was reported in only one study [52]. All but one study [64] clearly defined athlete demographics and outcome variables. Six studies did not include information surrounding familiarisation of the athletes to the COD task to be performed [48–51, 59, 88]. Eight [50, 51, 58, 59, 62–64, 77] of the 17 included studies conducted inappropriate statistical processes when examining differences between males and females in COD biomechanics, through pooling data across tasks [50, 62–64], maturation [51], fatigue [58, 77] and bracing [59] protocols prior to performing statistical analysis.

### Quantitative Synthesis: Kinematics

#### Hip

**Fig. 2:**
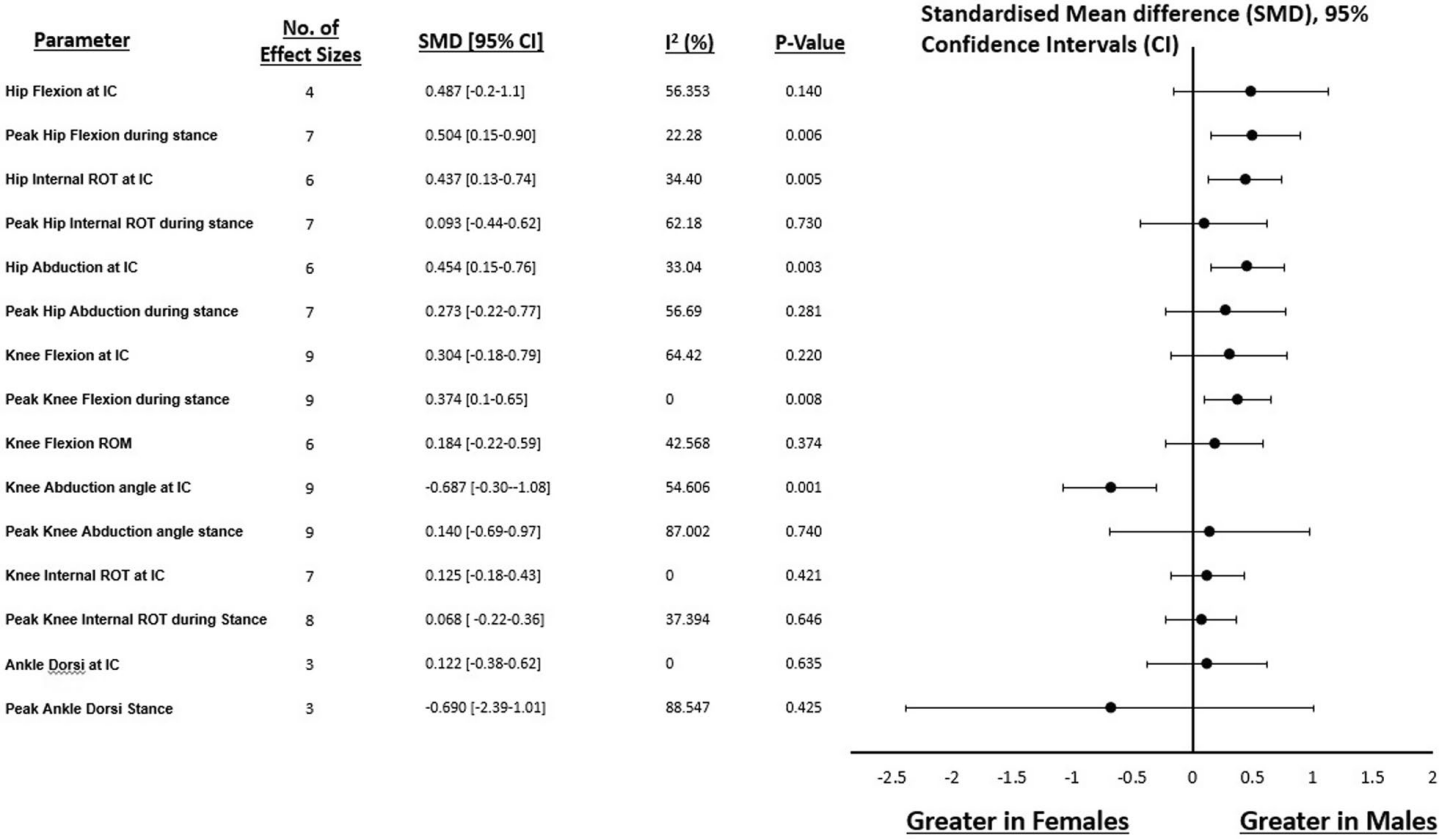
Forest plot illustrating standardised mean differences and 95% confidence intervals for determinants of KJLs between males and females. Key: Dorsi—Dorsiflexion, INT—Internal, IC—Initial contact, ROM—Range of motion, ROT—Rotation

In the sagittal plane, meta-analysis indicated that females exhibited less peak hip flexion during stance than males (SMD: 0.504, 95% CI 0.134–0.741, *p* = 0.06 I^2^: 22%; 7 measured groups with 155 participants (78 males, 77 females)) (Fig. 2). No statistical differences were found in hip flexion at initial contact (SMD: 0.487 95% CI −0.160 to 1.133, *p* = 0.140, I^2^: 56%; 4 studies with 93 participants (47 males, 46 females)) (Fig. 2). In the frontal plane, it was revealed that females produced significantly less hip abduction at initial contact (SMD: −0.454, 95% CI 0.151–0.758, *p* = 0.003, I^2^: 33%; 4 studies (two with multiple angle conditions) with 176 participants (93 males, 83 females)) (Fig. 2). No significant statistical differences in peak hip abduction during stance were observed (SMD: 0.273, 95% CI 0.223- 0.770, *p* = 0.281, I^2^: 57%; 7 measured groups with 156 participants (78 males, 78 females)) (Fig. 2). In the transverse plane, meta-analysis identified females produced significantly less internal rotation at initial contact (SMD: 0.437, 95% CI 0.134–0.741, *p* = 0.005, I^2^: 34%; 6 measured groups with 176 participants (93 males, 83 females)) while no statistical difference was found for peak hip internal rotation during stance (SMD: 0.093, 95% CI −0.436 to 0.623, *p* = 0.730, I^2^: 62%; 7 measured groups with 156 participants (78 males, 78 females)) (Fig. 2).

#### Knee

In the sagittal plane, meta-analysis indicated that females produced significantly less peak knee flexion during stance than males (SMD: 0.374, 95% CI 0.098–0.649), *p* = 0.008, I^2^: 0%; 9 measured groups with 209 participants (105 males, 104 females)), while no statistical differences were found for knee flexion at initial contact (SMD: 0.304, 95% CI 0.098–0.649, *p* = 0.220, I^2^: 64%; 9 measured groups with 204 participants (102 males, 102 females)) (Fig. 2). No statistical difference was found for knee flexion range of motion (SMD: 0.184, 95% CI −0.222 to 0.591, *p* = 0.374, I^2^: 43%; 6 measured groups with 169 participants (78 males, 91 females)) (Fig. 2). In the frontal plane, females displayed significantly greater knee abduction angles at initial contact (SMD: 0.687, 95% CI 0.299–1.076, *p* = 0.001, I^2^: 55%; 9 measured groups with 259 participants (134 males, 125 females)), but no statistical difference was found for peak knee abduction angle during stance (SMD: 0.140, 95% CI 0.690–0.971, *p* = 0.740, I^2^: 87%; 9 measured groups with 206 participants (103 males, 103 females)) (Fig. 2). In the transverse plane, knee rotation at initial contact and peak rotation during stance both indicated no statistical differences between males and females (SMD: 0.125, 95% CI −0.179 to 0.428, *p* = 0.421, I^2^: 0%; 7 measured groups with 169 participants (84 males, 85 females; (SMD: 0.068, 95% CI −0.224 to 0.360, *p* = 0646, I^2^: 37%; 8 measured groups with 186 participants (93 males, 93 females respectively)) (Fig. 2).

#### Ankle

In the sagittal plane, no statistical differences were observed between males and females at initial contact (SMD: 0.122, 95% CI −0.380 to 0.623, *p* = 0.623, I^2^: 0%; 3 measured groups with 62 participants (31 males, 31 females) and peak values during stance (SMD: 0.690, 95% CI -2.388–1.008, *p* = 0.425, I^2^: 89%; 3 measured groups with 62 participants (31 males, 31 females)) (Fig. 2).

#### Trunk

Insufficient evidence was synthesised to perform quantitative analysis with only one study [61] reporting outcome measures related to the trunk (forward and lateral inclination).

### Quantitative Synthesis: Kinetics

#### Knee Joint Loads

**Fig. 3:**
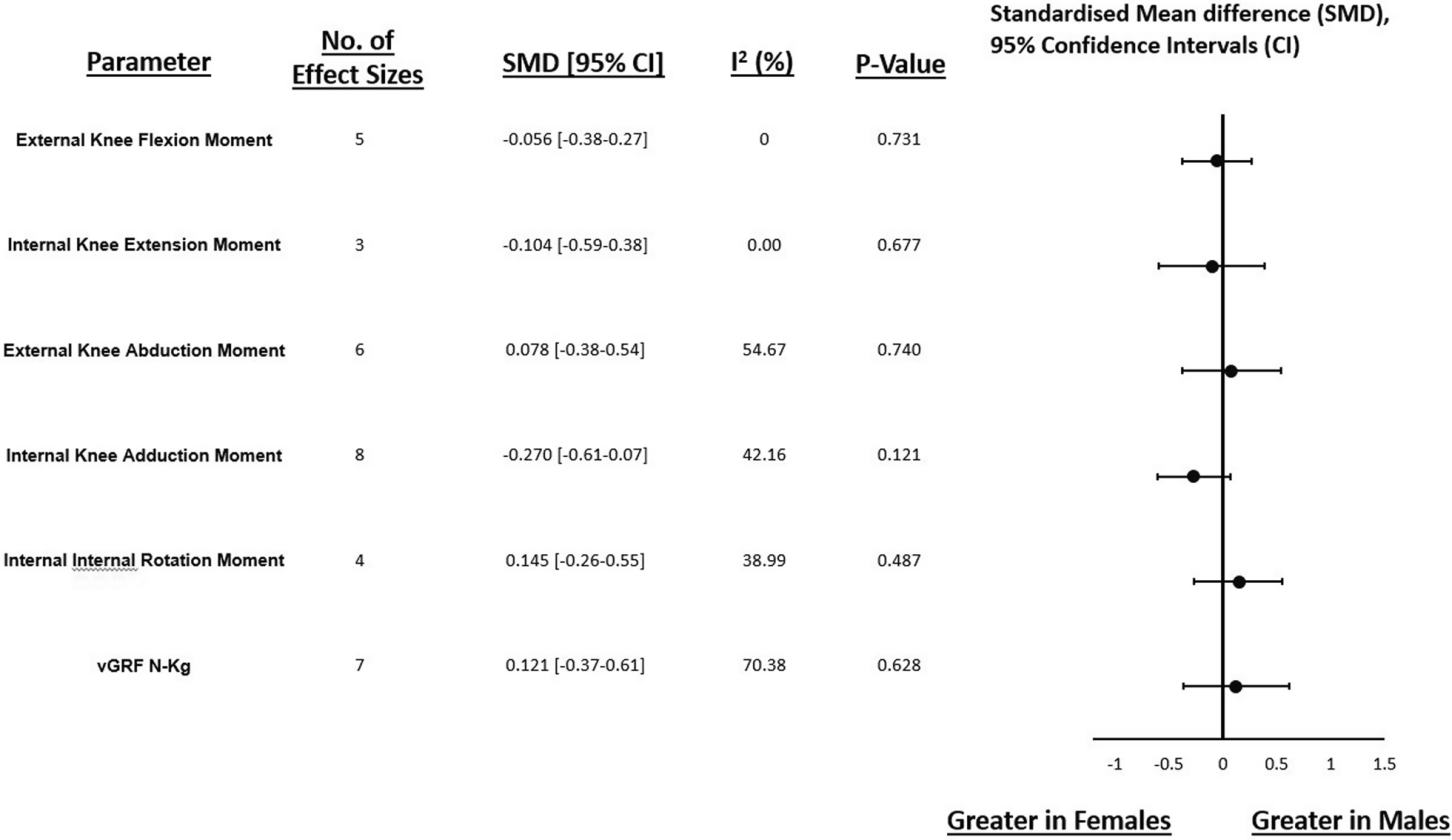
Forest plot illustrating standardised mean differences and 95% confidence intervals for multiplanar KJLs between males and females. Key: N-kg—Newtons per kilogram, vGRF—Vertical ground reaction force

In the sagittal plane, meta-analysis revealed no statistical differences between males and females for externally applied peak knee flexion moment (SMD: −0.056, 95% CI −0.379 to 0.266, *p* = 0.731, I^2^: 0%; 5 measured groups with 150 participants (69 males, 81 females)) and internal resultant knee extension moments (SMD: 0.104, 95% CI −0.592 to 0.384, *p* = 0.677, I^2^: 0%; 3 measured groups with 65 participants (32 males, 33 females)) (Fig. 3).

Regarding frontal plane knee moments, no statistical effect was observed between males and females for externally applied peak knee abduction moments (SMD: 0.078, 95% CI −0.382 to 0.537, *p* = 0.740, I^2^: 55%; 6 measured groups with 170 participants (79 males, 91 females)) and peak internal resultant knee adduction moments (SMD: −0.270, 95% CI −0.611 to 0.07, *p* = 0.121, I^2^: 42%; 8 measured groups with 249 participants (129 males, 120 females) (Fig. 3). Meta-analysis of transverse plane kinetics indicated no statistical effect of sex on internal resultant peak knee internal rotation moments (SMD: 0.145, 95% CI −0.264 to 0.553, *p* = 0.487, I^2^: 39%; 4 measured groups with 95 participants (47 males, 48 females)) (Fig. 3).

#### Ground Reaction Force

No statistical differences between males and females were observed for vertical GRF (SMD: 0.121, 95% CI −0.369 to 0.611, *p* = 0.628, I^2^: 70%; 7 measured groups with 232 participants (116 males, 116 females)) (Fig. 3).

### Subgroup and Moderator Analysis

Post hoc subgroup and moderator analyses were performed on variables that produced significant differences between males and females in the primary meta-analysis as follows: sagittal (peak during stance), frontal and transverse (initial contact) hip kinematics, together with sagittal (peak during stance) and frontal (initial contact) plane knee kinematics.

#### Hip Flexion During Peak Stance

Subgroup analysis for anticipated vs unanticipated CODs revealed no statistically significant difference between males and females in peak hip flexion during stance;(anticipated SMD: 0.809, 95% CI 0.345–1.274, I^2^ 0%; Z = 3.414 *p* = 0.001, ES = 4, 8 measured groups, 78 participants (39 males, 39 females); Unanticipated: SMD: 0.214, 95% CI −0.237 to 0.666, I^2^ = 24%, Z = 0.930, *p* = 0.352, ES = 3; 6 measured groups, 77 participants (39 male, 38 female)); Total between group analysis: Q = 3.233, *p* = 0.072. COD angle sub analysis was not possible due to the lack of data on COD angles other than 45°.

Subgroup analysis for sporting code revealed no statistical differences between males and females (soccer and basketball athletes) for peak hip flexion during stance;(soccer SMD: 0.194, 95% CI −0.237 to 0.625, I^2^ = 0%, Z = 0.883, *p* = 0.377, ES = 3; 6 measured groups, 84 participants (42 males, 42 females); basketball SMD: 0.880, 95% CI 0.149–1.612, I^2^ = 1%, Z = 2.358, *p* = 0.018, ES = 2; 4 measured groups, 32 participants (16 males, 16 females)); Total between group analysis: Q = 2.508, *p* = 0.113.

#### Hip Internal Rotation at Initial Contact

Subgroup analysis for anticipated vs unanticipated CODs revealed no statistically significant difference between males and females in hip internal rotation at initial contact;(anticipated SMD: 0.343, 95% CI −0.197 to 0.882, I^2^ = 59%, Z = 1.245, *p* = 0.213, ES = 3; 6 measured groups, 56 participants (28 males, 28 females); unanticipated SMD: 0.481, 95% CI 0.115–0.848, I^2^ = 24%, Z = 2.573, *p* = 0.010, ES = 3; 6 measured groups, 120 participants (65 males, 55 females);Total Between analysis: Q = 0.174, *p* = 0.677. Insufficient data were present on other COD angles to perform subgroup analysis.

Subgroup analysis for sporting code revealed no statistical differences between males and females in hip internal rotation at initial contact;(soccer SMD: 0.398, 95% CI 0.064–0.731, I^2^ = 20%, Z = 2.339, *p* = 0.019, ES = 4; 8 measured groups, 144 participants (77 males, 67 females); basketball SMD: 0.651, 95% CI219–1.520, I^2^ = 72%, Z = 1.685, *p* = 0.142, ES = 2; 4 measured groups, 32 participants (16 males, 16 females)); Total between analysis: Q = 0.237, *p* = 0.626.

#### Hip Abduction at Initial Contact

Analysis for the effect of anticipation revealed no statistical differences between sexes in hip abduction at initial contact (anticipated SMD: −0.067, 95% CI −0.600 to 0.446, I^2^ = 46%, Z = −0.245, *p* = 0.806, ES = 3; 6 measured groups, 56 participants (28 males, 28 females); unanticipated SMD: −0.640, 95% CI −1.010 to −0.271, I^2^ = 0%, Z = -3.401, *p* = 0.001, ES = 3; 6 measured groups, 120 participants (65 males, 55 females)) Total between group analysis: Q = 3.010, P = 0.083. Insufficient data were available to perform COD angle dependent analysis.

Sport subgroup analysis revealed a significant difference between soccer and basketball, with male soccer athletes favouring greater levels of hip abduction at initial contact (soccer SMD: −0.643 95% CI −0.980 to −0.307, I^2^ = 0%, Z = -3.744, *p* < 0.001, ES = 4; 8 measured groups, 144 participants (77 males, 67 females); basketball SMD: 0.362 (95% CI −0.338 to 1.062, I^2^ = 0%, Z = 1.013, *p* = 0.311, ES = 2; 4 measured groups, 32 participants (16 males, 16 females); Total between group analysis: Q = 6.430, *p* = 0.011.

#### Knee Flexion During Stance

Subsequent analysis indicated anticipation had no statistical effect on the amount of peak knee flexion observed between males and females during the stance phase of the COD (anticipated SMD: 0.433, 95% CI 0.114–0.753, I^2^ = 0%, Z = 2.659, *p* = 0.008, ES = 7; 156 participants (78 males, 78 females); unanticipated SMD: 0.200, 95% CI −0.343 to 0.744, I^2^ = 28%, Z = 0.722, *p* = 0.471, ES = 2; 53 participants (27 males, 26 females)); Total between group analysis: Q = 0.526, *p* = 0.468. Insufficient data were present for subgroup analysis on COD angle.

Subgroup analysis of sport revealed no statistical effect of sport on peak knee flexion angle between males and females during stance (soccer SMD: 0.311, 95% CI −0.122 to 0.744, I^2^ = 7%, Z = 1.406, *p* = 0.160, ES = 3; 6 measured groups, 84 participants (42 males, 42 females); basketball SMD: 0.803 95% CI 0.081–1.525, I^2^ = 0%, Z = 2.180, *p* = 0.029, ES = 2; 4 measured groups, 32 participants (16 males, 16 females); Physically active SMD: 0.213, 95% CI −0.236 to 0.663, I^2^ = 0%, Z = 0.929, *p* = 0.353, ES = 3; 6 measured groups, 77 participants (39 males, 38 females)); Total between analysis: Q = 1.900, *p* = 0.387.

#### Knee Abduction at Initial Contact

Subgroup analysis indicated no statistical effect of anticipation on knee abduction at initial contact between males and females (anticipated SMD: 0.656, 95% CI 0.068–1.245, I^2^ = 71%, Z = 2.185, *p* = 0.029, ES = 5; 10 measured groups, 106 participants (53 males and 53 females); unanticipated SMD: 0.726, 95% CI 0.153–1.298, I^2^ = 12%, Z = 2.485, *p* = 0.013, ES = 4; 8 measured groups, 153 participants (81 males, 72 females)); Total Between group analysis: Q = 0.028, P = 0.868.

Analysis of COD angle indicated no significant statistical effect on knee abduction angle between males and females at initial contact: 45° SMD: 0.613, 95% CI 0.073–1.152, I^2^ = 48%, Z = 2.226, *p* = 0.026, ES = 6; 12 measured groups, 178 participants (91 males, 87 females); 180° SMD: 0.936, 95% CI −0.120–1.993, I^2^ = 87%, Z = 1.737, *p* = 0.082, ES = 2; 4 measured groups, 36 participants (18 males, 18 fsemales):; Total Between group analysis: Q = 0.336, P = 0.845.

Sporting code subgroup analysis indicated a significant statistical effect of sport with female basketball players generating greater knee abduction angles at initial contact: soccer SMD: 0.650 95% CI 0.271–1.028, I^2^ = 37%, Z = 3.361, *p* = 0.001, ES = 5; 12 measured groups, 191 participants (102 males, 89 females); basketball SMD: 1.781, 95% CI 0.889–2.672, I^2^ = 0%, Z = 3.915, *p* < 0.001, ES = 2; 4 measured groups, 32 participants (16 males, 16 females); total between group analysis: Q = 5.239, *p* = 0.022.

#### Moderator Analysis

Moderator analysis for hip abduction angle at IC observed a significant statistical effect for age (mean male and female age modelled together: Q = 6.36, *p* = 0.0416), mass (mean male and female mass modelled together: Q = 6.49, *p* = 0.0390) and achieved velocity (mean male and female velocity modelled together: Q = 6.58, *p* = 0.0372). Moderator analysis for knee abduction angle at IC revealed a significant effect for methodological quality score (B = −0.1565, *p* = 0.03).

## Discussion

To the authors’ knowledge, this is the first meta-analysis that has examined multiplanar whole body kinematics and knee kinetics between males and females during COD tasks. No effect of sex on knee kinetics was identified despite extensive literature suggesting apparent sex differences in multiplanar KJLs in COD tasks [48, 50–52, 90], although females were identified as demonstrating greater knee abduction at IC and less peak knee flexion during stance. This is counterintuitive when considering the significant differences observed in sagittal and frontal plane knee motion for females that would increase the moment arm of the GRF vector in the relevant plane, and would amplify the KJLs generated by females [70]. Therefore there may be sex differences in the proportion of the KJL that is determined by the lever arm (lower limb) vs the GRF profile (ground impact profile). It is worth noting that the analysis of resultant internal knee adduction moment fell only marginally outside the 95% confidence interval levels for being greater in females (95% CI −0.611 to 0.07, *p* = 0.121), suggesting a potential undetected effect of sex. Considering a lack of differences in KJLs, it is feasible that these high-risk kinematics observed in females could have been offset by differences in the GRF profile (the other component of the KJL) whereby males produce more force resulting in comparable KJLs. This was not the case as meta-analysis revealed no significant differences between males and females for vertical GRF (*p* = 0.628). A small number of groups were measured in this analysis (seven), and there were only sufficient data present to analyse vertical GRF. Further research investigating this is required, incorporating analysis of multiplanar forces such as lateral GRFs. This would affect the moment arm of the force in the frontal plane during COD tasks and has been observed in a group of females exhibiting “excessive valgus” [44].

Males were identified as producing greater hip abduction and internal rotation at IC, identified as KJL determinants previously due to the intersegmental relationship between hip and knee positioning further down the kinetic chain [40, 44, 91]. A wider foot-plant would allow greater perpendicular forces to be produced achieving more effective task completion in more mechanically demanding CODs (> 60°) [92], in line with the faster performance times observed by males in the only study reporting performance time [63]. Interestingly, these hip postures did not translate to greater knee abduction angles in males compared to females despite the determinant relationship with KJLs [45]. This raises the question of other confounding factors such as pelvic width to femoral length ratios [93] being responsible for these knee postures in females. From the above, it is possible there are sex differences in the proportion of the KJL that is generated from lower limb kinematics vs the GRF profile, with females adopting more abducted and extended knee postures. It is worth considering that similar KJLs generated by males and females may not translate to similar injury risk, due to morphological differences in strain characteristics of the ACL between males and females [94, 95]. Therefore, the evidence concerning differences in KJLs and injury risk between males and females remains contentious and requires further investigation.

This meta-analysis indicates that sex has an effect on lower limb kinematics in COD tasks, namely multiplanar hip and knee kinematics. Females were found to execute COD tasks with greater knee abduction and less knee flexion. Considering the relationship between increased knee abduction and limited knee flexion with ACL strain [28, 30, 96], this observation may partially explain some of the disproportionately greater ACL injury incidence between females and males [19, 97, 98]. This finding also aligns with observational studies identifying limited knee flexion and dynamic knee valgus to be apparent features of non-contact ACL injury [9–11], particularly in females [10, 99–101].

Previous studies have examined differences between males and females in both landing [54], cutting [55] and weight-bearing tasks [53], although these results are somewhat inconclusive. Beaulieu et al. [54] identified females as landing with greater peak knee abduction angles than their male counterparts across a range of unilateral and bilateral drop-landings, although a lack of evidence was observed for knee joint loads and other lower limb kinematics. Benjaminse et al. [55] identified small differences between males and females during cutting, with females generating lower knee flexion angles and greater knee abduction angles and moments. However, they questioned the clinical relevance of these findings due to the lack of statistical power in the majority of included studies and inconsistent effect sizes. A limitation of these studies is the absence of any meta-analysis or regression that may identify differences between groups of males and females. Cronstrom et al. [53] detailed that females exhibited greater knee abduction and excursion across a range of weight bearing activities, including cutting, although this analysis only included frontal plane knee kinematics. Considering non-contact ACL injuries [102], ACL strain [96] and KJLs in CODs are multiplanar by nature and the result of a complex interaction of multiple body segments [45], this justified the current investigation of multiplanar kinematics and kinetics of the lower limb to further understand these potential differences between males and females.

In a number of these studies examining KJLs, data had been pooled across a number of conditions prior to performing sex comparisons [51, 59, 60, 63]. Such practice is questionable due to the confounding effects of task [50, 90], which has been previously identified as biomechanically discrete in relation to multiplanar knee joint loads and braking characteristics [103]. Furthermore, effects of maturation [51, 101], fatigue [60] and externally applied bracing [59] provide another layer of measurement variability that could skew the interpretation of sex differences in COD mechanics should the data be pooled prior to performing sex comparisons. The current findings of this analysis suggest that more carefully controlled research is required examining sex differences in COD biomechanics. Cronstrom et al. [104] identified knee abduction moments as not being predictive of non-contact ACL injuries in a recent meta-analysis despite contrary prospective findings in the literature [105, 106]. It is worth noting that this was across a range of screening tasks that predominantly consisted of drop landings, which have been identified as generating lower multiplanar KJLs compared to COD tasks [107]. Further prospective research is recommended explicitly in COD to identify the predictive utility of multiplanar knee joint loads in ACL injury. It still remains unexplained as to why there is a gynocentric pattern in non-contact ACL epidemiological literature, although a multifactorial approach that includes anatomical and hormonal contributions must be considered alongside multiplanar knee joint loads.

In addition, embedding a gendered, environmental approach [108] into these recommendations may provide further explanation for the discrepancy between males and females in ACL injury rates in multidirectional sports. This approach would account for sociocultural and socio-economic factors that could affect accumulated motor experience and resistance training history, explaining differences in motor skill and strength [109]. This could confound results when comparing males and females as previously stated [110], and lead to differences being attributed due to sex rather than a modifiable confounding variable. Evidence to support this can be seen in ballet, where there is a substantially lower ACL injury incidence (0.009 per 1000 exposures), and sex is not a risk factor for ACL injury [111]. This can be attributed to females receiving targeted training from an early age in high risk movements such as single legged landing, evidenced through comparable KJLs between males and females during this task [112]. Further investigation is required in multidirectional sports to confirm this notion, through matching participants for skill and resistance training history in COD studies examining KJLs. None of the retrieved studies in this meta-analysis controlled for motor skill levels/skill training history or examined differences in co-ordination, and future research should better account for these factors when examining biomechanical differences between males and females.

It is worth noting that none of the studies included in this analysis included a measurement of lower limb strength to serve as a control for sex comparisons. Hip abductor and external rotator strength have been identified as predictive of ACL injury in male and female populations [113], with pre-adolescent boys demonstrating greater hip external rotator strength, alongside greater gluteus medius activity in the pre-activation phase of a cutting task [114]. Increased lower limb strength, and eccentric strength in particular, would facilitate greater muscular support of the knee joint and maintain postural integrity through simultaneous co-activation of the hamstrings and quadriceps [115], potentially lowering the KJLs generated during CODs [39, 74]. This would also allow more effective utilisation of the penultimate foot-contact as a braking or preparatory step to also lower KJLs and improve performance times as previously demonstrated [39, 74]. Strength has also been identified as a determinant of COD mechanics [116, 117] and COD performance [118, 119]. Stronger individuals typically adopt a hip dominant strategy with greater levels of knee flexion [116, 117] and reduced internal knee extensor moments [117], thus lowering ACL strain [96]. Considering the above, this raises questions about the strength status of female participants in the retrieved studies, as meta-analysis identified females displaying reduced levels of knee flexion.

Therefore, without directly measuring strength qualities and thus, the absence of a strength control in the synthesised literature, it is postulated that relatively stronger males may have been compared with relatively weaker females, providing some potential explanation for these reported differences in KJLs. Collegiate female athletes participate less frequently in strength based training (1.9–3.0 vs 2.6–3.8 days per week) and for a shorter period of time (26–44 min per week vs 49–70 min per week) [120]. This could be explained from a gendered perspective whereby there are negative societal expectations concerning the appropriateness of muscular strength training in females [108, 121–123]. Therefore, as strength is a modifiable risk factor which can be targeted and trained, the relationship between female participation in strength training, strength, and multiplanar KJLs warrants further investigation to provide further insight about potential sex differences in COD biomechanics and ACL injury rates.

This meta-analysis explored sex differences in multiplanar knee joint loads (KJLs) and whole-body kinematics during change-of-direction (COD) tasks. The study extracted data from 17 retrieved articles involving 451 participants. Recommendations include accounting for resistance training history, motor skill experience and co-ordination, and gender-environmental factors in future research. Considering menstrual cycle phase together with variability and reliability is imperative when examining differences in males and females, due to measures of reliability being reported for only two studies [52, 57] and only one study controlling for menstrual cycle phase [56]. A range of COD angles were used in the included studies. While most data were extracted from 45° CODs and data from other angles pooled, this may not fully examine biomechanical sex differences at larger angles (≥ 90°). However, no significant angle-related effects were observed in the subgroup analysis. All data extracted were the product of discrete point analysis, which captures peak values for injury surrogates such as knee abduction angle or moment. However, this approach overlooks the complexity of COD, which involves multiple phases (approach run, deceleration, redirection, and re-propulsion). By focusing on a single data point, 99% of the remaining cutting cycle is discarded [36]. To gain deeper insights, future research should explore non-contact ACL injury risk using statistical parametric mapping methods, considering the entire cutting cycle. This comprehensive approach would enhance our understanding of potential differences between males and females in COD tasks.

Finally, from the findings of this meta-analysis we cannot discern underlying reasons for observed sex differences in COD biomechanics. Other biomechanical, anatomical and hormonal factors such as quadriceps dominance [124], pelvic width to femoral length ratio [93] and fluctuations in serum sex hormones such as oestrogen in the pre-ovulatory phase [125] all likely interact and may contribute to observed differences in surrogates of non-contact ACL injury risk. Furthermore, there was a failure to examine any differences in co-ordination, resistance or skill training history. It is likely these factors contribute to these sex differences, especially when observing these variables through the lens of a gendered environmental approach [108]. Socioeconomic and gender related factors should be acknowledged regarding their contribution to motor skill and strength levels [110]. Further research incorporating all of these modifiable and non-modifiable risk factors is recommended together with better reporting of female demographics [126] to further understand the interaction and respective contributions these have to COD multiplanar KJLs.

## Conclusion

This systematic review and meta-analyses revealed no differences between males and females in multiplanar KJLs despite apparent differences between males and females in COD kinematics. Further research is required to identify if this translates to similar injury risk, considering morphological differences in strain characteristics between males and females. Females exhibit increased knee abduction and limited hip and knee flexion compared to males, whereas males display increased hip abduction and internal rotation. Considering the reported difference in knee abduction and flexion in CODs, there is a need to focus on this movement pattern during COD technique modification programmes in females. All of the included studies included healthy males and females, predominantly of a collegiate or recreational background, therefore further research is required in ACL deficient and elite populations with considerations for socioeconomic factors, skill training history and strength levels.

## Data Availability

Not applicable.

## Abbreviations

3D: Three dimensional
ACL: Anterior cruciate ligament
COD: Change of direction
GRF: Ground reaction force
HC: Hormonal contraception
I^2^: I squared
IC: Initial contact
MSK: Musculoskeletal
KJL: Knee joint load
SD: Standard deviation
SMD: Standardised mean difference
